# EGFR signaling pathway occupies an important position in cancer‐related downstream signaling pathways of Pyk2

**DOI:** 10.1002/cbin.11209

**Published:** 2019-08-11

**Authors:** Ting Shen, Qiang Guo

**Affiliations:** ^1^ Medical School Kunming University of Science and Technology Kunming 650500 Yunnan China; ^2^ Department of Gastroenterology, The Affiliated Hospital of Kunming University of Science and Technology The First People's Hospital of Yunnan Province Kunming 650032 Yunnan China

**Keywords:** cancer, cell migration, intercellular communication, signal peptide/recognition particle

## Abstract

Proline‐rich tyrosine kinase 2 (Pyk2) is a member of focal adhesion kinase (FAK) non‐receptor tyrosine kinase family and has been found to promote cancer cell survival, proliferation, migration, invasion, and metastasis. Pyk2 takes part in different carcinogenic signaling pathways to promote cancer progression, including epidermal growth factor receptor (EGFR) signaling pathway. EGFR signaling pathway is a traditional carcinogenic signaling pathway, which plays a critical role in tumorigenesis and tumor progression. FAK inhibitors have been reported to fail to get the ideal anti‐cancer outcomes because of activation of EGFR signaling pathway. Better understanding of Pyk2 downstream targets and interconnectivity between Pyk2 and carcinogenic EGFR signaling pathway will help finding more effective targets for clinical anti‐cancer combination therapies. Thus, the interconnectivity between Pyk2 and EGFR signaling pathway, which regulates tumor development and metastasis, needs to be elucidated. In this review, we summarized the downstream targets of Pyk2 in cancers, focused on the connection between Pyk2 and EGFR signaling pathway in different cancer types, and provided a new overview of the roles of Pyk2 in EGFR signaling pathway and cancer development.

**Abbreviations**
AR: androgen receptor
Arg: Abl‐related gene
BCSC: breast cancer stem cell
CCL19: chemokine (C‐C motif) ligand 19
CCR7: chemokine receptor 7
CREB: cyclic‐AMP response element‐binding protein
DFX: deferasirox
ECM: extracellular matrix
EGFR: epidermal growth factor receptor
EMT: epithelial‐mesenchymal transition
ERK: extracellular regulated protein kinase
FAK: focal adhesion kinase
FAT: focal adhesion targeting
GSTO1: glutathione S‐transferase omega 1
HER2: human epidermal growth factor receptor 2
Hic‐5: hydrogen peroxide inducible clone‐5
HRG: heregulin
JAK: janus kinase
MAPK: mitogen‐activated protein kinase
MM: multiple myeloma
MMP‐10: matrix metalloproteinase‐10
NDRG1: N‐myc downstream regulated 1 gene
NEDD4: neural precursor cell‐expressed developmentally downregulated gene 4
PCa: prostate cancer
pEGFR: phosphorylated epidermal growth factor receptor
pERK: phosphorylated extracellular signal‐regulated protein kinase
PI3K: phosphoinositide 3‐kinase
PKC: protein kinase C
PTEN: phosphatase and tensin homolog deleted on chromosome ten
PTK: protein tyrosine kinase
Pyk2: proline‐rich tyrosine kinase 2
RTKs: receptor tyrosine kinases
S6K: S6‐kinase
SCCHN: squamous cell carcinoma of the head and neck
SCLC: small cell lung cancer
STAT: signal transducers and activators of transcription
TNBC: triple negative breast cancer
TNFRSF19/TROY: mouse tumor necrosis factor receptor superfamily member 19
VEGF: vascular endothelial growth factor

## Introduction

Focal adhesion kinase (FAK), a kind of multi‐domain non‐receptor protein tyrosine kinase (PTK), controls cell survival, adhesion, and migration by transferring signals from integrins or growth‐factor receptors to downstream kinases (Arold, 2011; Kleinschmidt and Schlaepfer, 2017). FAK is widely detectable in adult tissues and aberrant expression of FAK could be regarded as a promising factor to predict aggressive behavior and poor prognosis in patients with tumors (Ji et al., 2013; Li et al., 2015; Omura et al., 2016). FAK is overexpressed in many kinds of tumors and contributes to tumorigenicity and tumor development (Sood et al., 2004; Carelli et al., 2006; Yom et al., 2011; Tai et al., 2016). Proline‐rich tyrosine kinase 2 (Pyk2) is a close paralogue to FAK and possesses 46% sequence identity and 65% similarity related to FAK in structure (Du et al., 2001; Schaller, 2010). The effects on cellular events are not always the same between FAK and Pyk2. Pyk2 is only abundant in specific cell types such as macrophages, osteoclasts, and lymphocytes (Menegon et al., 1999; Allen et al., 2009; Beinke et al., 2010; Gao et al., 2015) and could be activated by multiple growth factors, neuropeptides, cytokines, hormones, and chemokines (Ivankovic‐Dikic et al., 2000; Di Cioccio et al., 2004; Roelle et al., 2008; Cattaneo et al., 2009; Lane et al., 2016). In numerous researches in vivo and in vitro, overexpression of Pyk2 is found in different malignant tumors (Sun et al., 2007; Zhang et al., 2008; Hsiao et al., 2016) and it is implicated in multiple signal transduction cascades, which regulate cancer cell proliferation, apoptosis, and invasion (Okigaki et al., 2003; Sun et al., 2008; Wiese et al., 2015). Pyk2 promotes tumor progression and owes to a number of cancer‐related functional domains in structure: N‐terminal FERM domain, a central catalytic kinase domain, and C‐terminal focal adhesion targeting (FAT) domain (Lipinski and Loftus, 2010). The FERM domain of Pyk2 could mediate both protein‐protein and protein‐membrane targeting interactions and plays a critical role in Pyk2‐induced migration of tumor cells (Hirao et al., 1996; Hamada et al., 2000, 2003; Pearson et al., 2000; Loftus et al., 2009). Pyk2 contains central catalytic kinase domain that may be of potential use in the design of selective kinase inhibitors for cancer treatments (Han et al., 2009). The C‐terminal domain of Pyk2 includes a FAT domain, which is implicated in the activation of carcinogenic mitogen‐activated protein kinase (MAPK) signaling pathway (Blaukat et al., 1999; Kuang et al., 2013). In recent years, Pyk2 is found to be involved in epidermal growth factor receptor (EGFR) signaling pathway in cancer progression.

EGFR is a member of the EGF receptor tyrosine kinase family, which includes EGFR (ErbB1/HER1), HER2/neu (ErbB2), HER3 (ErbB3), and HER4 (ErbB4). EGFR is a transmembrane growth factor receptor and its downstream signaling pathways frequently contribute to tumor progression and metastasis (Ciardiello and Tortora, 2008; Kumar et al., 2016; Koustas et al., 2017; Singla et al., 2018). EGFR signaling mainly contains the RAS/MEK/ERK (extracellular regulated protein kinase), PI3K (phosphoinositide 3‐kinase)/AKT and PLCγ/PKC (protein kinase C) cascades, moreover, the Src tyrosine kinase and janus kinase (JAK)/signal transducers and activators of transcription (STAT) pathway are also induced by EGFR activation (Brand et al., 2011). After ligand binding to EGFR, receptor auto‐transphosphorylation triggers a series of signaling events, which result in the induction of cell proliferation, blockade of apoptosis, activation of invasion, and stimulation of neovascularization (Shepard et al., 2008). With ligand combining with EGFR, STAT3 is phosphorylated and promotes tumor cell invasion and poor prognosis of colorectal adenocarcinoma (Kusaba et al., 2006). Overexpression of EGFR antagonizes neoalbaconol‐induced VEGF reduction and impairs anti‐angiogenesis of neoalbaconol in cancer (Yu et al., 2017). EGFR‐related downstream proteins, such as phosphatase and tensin homolog deleted on chromosome ten (PTEN), PI3K, and Akt, could have a significant impact on cell proliferation or apoptosis (Harle et al., 2015). EGFR can activate the RAS‐MEK‐ERK pathway and lead to cell proliferation and survival, which makes it a suitable target for cancer inhibition (Misale et al., 2014).

Pyk2 and EGFR signaling pathway are both proved to decide the fate of cancer. However, the exact role that Pyk2 plays in the EGFR signaling pathway still remains unclear. Traditionally, Pyk2 has been identified as a potential therapeutic target for human cancer treatment. Targeting Pyk2 could regulate its downstream signaling pathways and control the growth and metastasis of cancer cells. However, monotherapy of FAK has been found to fail to get the ideal anti‐cancer outcomes because of the effects of compensatory signaling. Thus, as a member of the FAK family, Pyk2‐related network of tumorigenesis and tumor progression needs to be elucidated and Pyk2‐related carcinogenic signaling pathways should be paid more attention to. EGFR signaling has an important place and role in Pyk2 downstream signaling pathways, which will affect the growth and metastasis of cancer cells. In this review, we summarize the recent findings of endogenous mechanisms used by cells with respect to Pyk2‐related regulation of cancer cell growth, proliferation, apoptosis, migration, invasion, metastasis, tumorigenesis, and tumor angiogenesis. We explore the interconnectivity between Pyk2 and EGFR signaling pathway in different cancer types, as well as aid in the identification of potential targets for cancer therapy. A systematic understanding of these mechanisms could contribute to the design of novel and more effective therapeutic interventions, which will block the aggressive growth of cancer cells.

## Anti‐cancer effectiveness of FAK inhibitors could be arrested by compensatory EGFR‐related signaling

FAK is overexpressed in 80% of all solid tumors and FAK inhibitors have been considered as promising anti‐cancer drugs (Weiner et al., 1993; Owens et al., 1995; Lark et al., 2003). However, anti‐cancer clinical trials of FAK inhibitors show the limited single‐agent efficacy (Gan et al., 2012; Infante et al., 2012) and compensatory signaling has been found to be responsible for this phenomenon. In the study performed by Marlowe et al., the results confirmed that the expression of receptor tyrosine kinases (RTKs) predicted patient response to FAK‐kinase inhibitors. FAK‐kinase inhibition induced RTK activation in RTK high cancer cells while the selective pressure of FAK‐kinase inhibition was able to drive RTK low triple‐negative breast cancer cells to express human epidermal growth factor receptor 2 (HER2). The inhibition of FAK induced compensatory increases of phosphorylated EGFR (pEGFR), pHER2, pAKT, and phosphorylated extracellular signal‐regulated protein kinase (pERK). Moreover, FAK inhibition led to a paradoxical increase of phosphorylated FAK tyrosine 397 (FAK pY397), which could maintain FAK‐dependent cancer cell migration and invasion. HER2 (and potentially other RTKs) was found to bind to FAK FERM F1 lobe and promoted its proximity to FAK Y397, allowing HER2 to directly phosphorylate FAK Y397 (Marlowe et al., 2016). In addition, E‐cadherin has been reported to arrest FAK inhibitor‐induced apoptosis and support merlin‐negative malignant mesothelioma progression (Kato et al., 2017). ALDH1A3, CD44, and MDR1 were found to play a role in the cancer cell resistance to FAK autophosphorylation inhibitor Y15 and their downregulation would sensitize resistant cancer cells to Y15 (Golubovskaya et al., 2015).

## Pyk2 promotes different cancer progression by modulating distinct downstream target sites

In multiple kinds of cancer types, Pyk2 mediates its downstream target genes and controls a variety of signaling pathways that are involved in human cancer cell growth, proliferation, apoptosis, migration, invasion, and metastasis, as well as tumorigenesis and tumor angiogenesis (Tables 1 and 2). EGFR signaling occupies an important position among these signaling pathways.

**Table 1:** Downstream target sites of Pyk2 in human cancers. Pyk2 increases the activation of some downstream targets to reinforce human cancer progression and invasion

Target sites	Cancer types	Involved cell lines	The biological roles of downstream targets of Pyk2 in cancers	References
CREB	Neuroblastoma	SH‐SY5Y cell	Promoting the viability of tumor cells	Hirschler‐Laszkiewicz et al. (2018)
ALDH1a1	Lung cancer	A549 cell, H460 cell	Enhancing cancer cell colony formation	Kuang et al. (2013)
ABCG2	Lung cancer	A549 cell, H460 cell	Augmenting cancer cell colony formation	Kuang et al. (2013)
Bmi‐1	Lung cancer	A549 cell, H460 cell	Promoting cancer cell colony formation	Kuang et al. (2013)
HER3	Breast cancer	MDA‐MB‐468 cell, HCC38 cell, HCC1143 cell, BT‐20 cell, HCC1937 cell	Augmenting cancer cell growth, survival, and proliferation	Verma et al. (2017)
Paxillin	Multiple myeloma	MM.1 S cell	Promoting cell‐cycle progression, adhesion ability, and proliferation of tumor cells	Zhang et al. (2014)
Rac1	Glioma	T98G cell	Promoting tumor cell migration	Paulino et al. (2010)
N‐Cadherin	Liver cancer	MHCC97L cell	Facilitating EMT, motility, and migration of cancer cells	Sun et al. (2011)
Hic‐5	Liver cancer	Hep3B cell, MHCC97L cell	Promoting EMT, motility, and migration of cancer cells	Sun et al. (2011)
STAT5b	Liver cancer	MHCC97L cell	Promoting EMT, motility, and migration of cancer cells	Sun et al. (2011)
p130 Cas	Breast cancer	MDA‐MB‐468 cell, MDA‐MB‐231 cell, MDA‐MB‐435scell, MDA‐MB‐453 cell, and MCF‐7 cell	Enhancing migration and invasion of cancer cells	Vultur et al. (2008)
AMAP1	Breast cancer	MCF‐7 cell	Promoting cancer cell adhesion, migration, and invasion	Li et al. (2018)
c‐Met	Breast cancer	MDA‐MB‐468 cell, BT‐549 cell	Promoting EMT, migration, invasion, and metastasis of breast cancer cells	Verma et al. (2015)
CD44	Breast cancer	MDA‐MB‐468 cell	Promoting EMT, migration, invasion, and metastasis of cancer cells	Verma et al. (2015)
Zeb‐1,2	Breast cancer	MDA‐MB‐468 cell, BT‐549 cell	Promoting EMT, migration, invasion, and metastasis of cancer cells	Verma et al. (2015)
Snail‐1,2	Breast cancer	MDA‐MB‐468 cell, BT‐549 cell	Promoting EMT, migration, invasion, and metastasis of cancer cells	Verma et al. (2015)
MMP	Breast cancer	BT‐549 cell, MDA‐MB‐231 cell, 2D cell	Promoting EMT, motility, migration, invasion, and metastasis of cancer cells	Verma et al. (2015); Genna et al. (2018)
Arg	Breast cancer	MDA‐MB‐231 cell, 2D cell	Promoting cancer cell motility, migration, and invasion	Genna et al. (2018)
Cortactin	Breast cancer	MDA‐MB‐231 cell, 2D cell	Enhancing cancer cell motility, migration, and invasion	Genna et al. (2018)
EGFR	Breast cancer	MDA‐MB‐468 cell	Promoting EMT, migration, invasion, and metastasis of cancer cells	Verma et al. (2015)
Fibronectin	Liver cancer; breast cancer	MHCC97L cell; MDA‐MB‐468 cell	Enhancing EMT, motility and migration of liver cancer cells; promoting EMT, migration, invasion, and metastasis of breast cancer cells	Sun et al. (2011); Verma et al. (2015)
Twist	Liver cancer; breast cancer	MHCC97L cell; MDA‐MB‐468 cell	Promoting EMT, motility, and migration of liver cancer cells; enhancing EMT, migration, invasion, and metastasis of breast cancer cells	Sun et al., 2011; Verma et al. (2015)
Vimentin	SCCHN; breast cancer	PCI‐4B cell, PCI‐37B cell; BT‐549 cell, MDA‐MB‐468 cell	Enhancing EMT, migration, invasion of SCCHN cells; promoting breast cancer cell EMT, migration, invasion, and metastasis	Verma et al. (2015); Yue et al. (2015)
FAK	Prostate cancer	PC3 cell	Augmenting cancer cell motility, invasion, and metastasis	Iiizumi et al. (2008)
MAPK	Prostate cancer	PC3 cell	Enhancing cancer cell motility, invasion, and metastasis	Iiizumi et al. (2008)
p90RSK	Bladder cancer	5637 cell	Promoting motility, migration, and invasion of bladder cancer cells	Genua et al. (2012)
MEK1/2	Liver cancer	PLC cell	Promoting proliferation and invasiveness of cancer cells	Sun et al. (2008)
β‐Catenin	Breast cancer; multiple myeloma	MDA‐MB‐468 cell; MM1S cell, RPMI8226 cell	Promoting EMT, migration, invasion, and metastasis of breast cancer cells; enhancing cell‐cycle progression, adhesion ability, and proliferation of multiple myeloma cells; inhibiting multiple myeloma cell apoptosis	Zhang et al. (2014); Verma et al. (2015); Kamihara et al. (2016)
Src	Lung cancer; multiple myeloma; breast cancer; liver cancer	H69 cell, H510 cell; MM.1 S cell; MDA‐MB‐231 cell; PLC cell	Enhancing lung cancer cell proliferation; promoting cell‐cycle progression, adhesion ability, and proliferation of multiple myeloma cells; promoting breast cancer cell motility, migration, and invasion; promoting proliferation and invasiveness of liver cancer cells	Roelle et al. (2008); Sun et al. (2008); Zhang et al. (2014); Lu et al. (2017); Genna et al. (2018)
AKT	Bladder cancer; prostate cancer; multiple myeloma; liver cancer	5637 cell; PC3 cell; MM.1S cell; PLC cell, Hep3B cell, MHCC97L cell, HL‐7702 cell, SMMC‐7721 cell, HepG2 cell	Promoting bladder cancer cell motility, migration, and invasion; augmenting prostate cancer cell motility, invasion, and metastasis; promoting cell‐cycle progression, adhesion ability, and proliferation of multiple myeloma cells; promoting liver cancer cell survival, growth, invasion, promoting liver cancer angiogenesis, arresting liver cancer cell apoptosis	Iiizumi et al. (2008); Geng et al. (2011); Genua et al. (2012); Cao et al. (2013); Zhang et al. (2014)
S6K	Bladder cancer, prostate cancer, breast cancer	5637 cell, T24 cell; LNCaP cell, 22Rv1 cell; MDA‐MB‐468 cell, HCC38 cell, HCC1143 cell, BT‐20 cell, HCC1937 cell	Promoting motility, migration, and invasion of bladder cancer cells; enhancing prostate cancer cell growth and proliferation; promoting breast cancer cell growth, survival, and proliferation, enhancing breast cancer growth	Genua et al. (2012); Hsiao et al. (2016); Verma et al. (2017)
STAT3	SCCHN; multiple myeloma; breast cancer	PCI‐4B cell, PCI‐37B cell; RPMI8226 cell, NCIH929 cell, OPM2cell; MDA‐MB‐468 cell, HCC38 cell, BT‐20 cell, HCC1937 cell	Promoting migration and invasion of SCCHN cells; promoting multiple myeloma cell survival; enhancing breast cancer cell growth, survival, proliferation, EMT, migration, invasion, and metastasis, promoting breast cancer growth	Liu et al. (2014); Verma et al. (2015); Meads et al. (2016); Verma et al. (2017)
ERK	Bladder cancer; prostate cancer; breast cancer; liver cancer; glioma	5637 cell, T24 cell; LNCaP cell, PC3 cell; MDA 231 cell, MDA‐MB‐435, MDA‐MB‐468 cell; PLC cell; C6 cell, SF767 cell	Promoting bladder cancer cell motility, migration, and invasion; promoting prostate cancer cell adhesion, proliferation, and differentiation; enhancing breast cancer cell proliferation, EMT, motility, migration, invasion, and metastasis; promoting proliferation and invasiveness of liver cancer cells; reinforcing glioma cell invasion	Zrihan‐Licht et al. (2000); Picascia et al. (2002); van der Horst et al. (2005); Yuan et al. (2007); Behmoaram et al. (2008); Sun et al. (2008); Genua et al. (2012); Verma et al. (2015)
GSK3β ^Y216^	Intestinal cancer	SW480 cell	Reinforcing tumorigenesis	Gao et al. (2015)

AKT, protein kinase B; CREB, cyclic‐AMP response element‐binding protein; EMT, epithelial‐mesenchymal transition; ERK, extracellular regulated protein kinase; FAK, focal adhesion targeting; HER3, human epidermal growth factor receptor 3; PLC, phospholipase C; MAPK, mitogen‐activated protein kinase; MDA, malondialdehyde; MMP, matrix metalloproteinases; Pyk2, proline‐rich tyrosine kinase 2; SCCHN, squamous cell carcinoma of the head and neck; S6K, S6‐kinase; STAT3, signal transducers and activators of transcription.

**Table 2:** Downstream target sites of Pyk2 in human cancers. Pyk2 promotes the progression of different cancers by inhibiting some downstream targets

Target sites	Cancer types	Involved cell lines	The biological roles of downstream targets of Pyk2 in cancers	References
Cytokeratin	Liver cancer	Hep3B cell; MHCC97L cell	Inhibiting EMT, motility, and migration of cancer cells	Sun et al. (2011)
E‐cadherin	SCCHN; breast cancer; liver cancer	PCI‐4B cell, PCI‐37B cell; BT‐549 cell, MDA‐MB‐468 cell; Hep3B cell, MHCC97L cell	Arresting EMT, migration, invasion of SCCHN cells; inhibiting breast cancer cell EMT, migration, invasion, and metastasis; inhibiting EMT, motility, and migration of liver cancer cells	Sun et al. (2011); Verma et al. (2015); Yue et al. (2015)
ZO‐1	Breast cancer	BT‐549 cell	Inhibiting cancer cell EMT, migration, invasion, and metastasis	Verma et al. (2015)
NDRG1	Breast cancer	MDA‐MB‐468 cell, HCC38 cell, HCC1143 cell, BT‐20 cell, HCC1937 cell	Inhibiting cancer cell growth, survival, and proliferation, attenuating cancer growth	Verma et al. (2017)

EMT, epithelial‐mesenchymal transition; NDRG1, N‐myc downstream regulated 1 gene; SCCHN, squamous cell carcinoma of the head and neck.

### Pyk2 regulates downstream targets and promotes cancer cell growth and proliferation

In hepatocellular carcinoma (HCC), Pyk2 overexpression increases MEK1/2 (mitogen‐activated protein kinase 1/2) phosphorylation, promotes the activation of c‐Src and ERK1/2, induces cancer cell proliferation (Sun et al., 2008). ERK is an important downstream target of Pyk2, which promotes cancer progression. Pyk2 contributes to ERK1/2 activation and enhances ErbB‐induced cell proliferation and breast cancer growth (Behmoaram et al., 2008). Pyk2 facilitates prostatic cancer cell proliferation by upregulating ERK1/2 phosphorylation (Picascia et al., 2002). Pyk2 depletion could inhibit the activation of S6K (S6‐kinase), STAT3, and HER3 while FAK depletion influences Akt activation in triple‐negative breast cancer (TNBC). Pyk2‐NDRG1 (N‐myc downstream regulated 1 gene)‐NEDD4 (neural precursor cell‐expressed developmentally downregulated gene 4) axis is proved to be a key regulator of HER3 degradation. Precluding Pyk2 could lead to the inhibition of TNBC cell growth, survival, and proliferation (Verma et al., 2017). Pyk2 activates ribosomal S6K1, regulates androgen receptor (AR) function, and enhances prostate cancer cell growth and survival (Hsiao et al., 2016). Cyclic‐AMP response element‐binding protein (CREB) is a nuclear transcription factor and regulates transcriptional responses to all kinds of growth factors and stress signals in cells (Shaywitz and Greenberg, 1999; Wang et al., 2016). Pyk2 downregulation is proved to decrease CREB phosphorylation and expression and inhibits the viability of neuroblastoma (Hirschler‐Laszkiewicz et al., 2018). Src is also a downstream target of Pyk2 in cancer development. Pyk2 promotes neuropeptide‐mediated Src kinase phosphorylation and neuropeptide‐stimulated survival and proliferation of small‐cell lung cancer (SCLC) cells while FAK activity isn't affected by neuropeptides in SCLC cells (Roelle et al., 2008). Pyk2 could induce the expression of cancer stem cell marker ALDH1a1, ABCG2, and Bmi‐1 and is proved to be associated with the colony formation of lung cancer cells (Kuang et al., 2013). In multiple myeloma (MM), Pyk2 plays a tumor‐promoting role and facilitates cell adhesion ability, cell‐cycle progression, and cell proliferation by activating Wnt/β‐catenin signaling. Inhibition of Pyk2 will result in the decrease of β‐catenin and p‐Akt. Moreover, Pyk2 overexpression is found to increase the phosphorylation of Src and Paxillin in MM cells (Zhang et al., 2014). Pyk2 shows a more malignant phenotype and promotes MM cell growth and proliferation by enhancing JAK1/STAT3 signaling (Meads et al., 2016). Pyk2 is important for cancer cell growth and proliferation.

### The roles of Pyk2‐modulated downstream targets in inhibiting cancer cell apoptosis

Overexpression of Pyk2 increases downstream AKT phosphorylation, arrests HCC cell necrosis and apoptosis, and contributes to cancer resistance to cisplatin (Geng et al., 2011). Iron chelator deferasirox (DFX) could inhibit Pyk2 expression, subsequently arrest β‐catenin expression, and induce MM cell apoptosis. However, FAK is not correlated with DFX‐induced MM cell apoptosis (Kamihara et al., 2016).

### Pyk2 enhances cancer cell migration, invasion, and metastasis by regulating different downstream targets

In the study of cancer cell migration, invasion, and metastasis, epithelial‐mesenchymal transition (EMT) has become an increasingly serious concern. EMT is the process that epithelial cells transit to invasive mesenchymal cells with epithelial genes downregulation and mesenchymal genes upregulation (Zeisberg and Neilson, 2009). Pyk2 promotes EMT by downregulating epithelial gene cytokeratin and E‐cadherin and upregulating mesenchymal gene Twist, N‐cadherin, fibronectin, hydrogen peroxide inducible clone‐5 (Hic‐5) and STAT5b, thus contributing to HCC cell motility and migration (Sun et al., 2011). In squamous cell carcinoma of the head and neck (SCCHN), E‐cadherin and vimentin are proved to be downstream target molecules of chemokine receptor 7 (CCR7)‐Pyk2, which may participate in the modulation of EMT, migration, and invasion of cancer cells (Yue et al., 2015). Inhibition of Pyk2 is reported to block the phosphorylation of STAT3 induced by chemokine (C‐C motif) ligand 19 (CCL19), thus arresting SCCHN cell EMT, migration, invasion, and metastasis (Liu et al., 2014). In breast cancer, Pyk2 is found to promote cancer cell migration, invasion, and metastasis by regulating distinct downstream targets. EGF activates Pyk2, regulates functions of downstream Twist‐1,2, CD44, Snail‐1,2, matrix metalloproteinase‐10 (MMP‐10), β‐catenin, fibronectin, vimentin, E‐cadherin, ZO‐1, and Zeb‐1,2, promotes EMT, migration, invasion, and metastasis of breast cancer cells. However, FAK cannot be activated by EGF. Under EGF stimulation, Pyk2, STAT3, and c‐Met interact with each other and form positive feedback, which contributes to prolonging EMT‐associated signals and cancer metastasis (Verma et al., 2015). Pyk2 has higher affinity with cortactin than FAK. Pyk2 colocalizes with cortactin to invadopodia of breast cancer cells and regulates EGF–induced cortactin phosphorylation through Src‐mediated Abl‐related gene (Arg) activation, leading to actin polymerization and breast cancer cell invasion. In addition, Pyk2‐depleted cells show a decreased MMP secretion and extracellular matrix degradation (Genna et al., 2018). The abundance of breast cancer stem cell (BCSC) has proved to be essential for breast cancer recurrence and metastasis. Pyk2/Src/STAT3 signaling pathway is activated by the rise of glutathione *S*‐transferase omega 1 (GSTO1)‐induced cytosolic calcium and leads to BCSC enrichment (Lu et al., 2017). Pyk2 acts downstream of ErbB‐2 and can be phosphorylated by heregulin (HRG). Phosphorylated Pyk2 activates ERK and plays a key role in breast cancer cell invasion (Zrihan‐Licht et al., 2000). Src/FAK/Pyk2/p130 Cas (crk‐associated substrate) is another effective pathway, which is reported to be associated with cell migration and invasion of breast cancer (Vultur et al., 2008). Through phosphorylating GTPase‐activating protein AMAP1, Pyk2 plays a critical role in CCL18‐induced cell adhesion, migration, and invasion in breast cancer (Li et al., 2018). ERK is an important downstream target, which contributes to different cancer cell migration, invasion, and metastasis. Overexpression of Pyk2 facilitates HCC cell invasiveness by upregulating the phosphorylation of c‐Src, ERK1/2, and MEK1/2 (Sun et al., 2008). Through activating Pyk2, elevated ErbB‐2 could increase the ERK/MAPK activity and enhance cell adhesive ability and metastasis in human prostate cancer (PCa), while FAK isn't correlated with PCa cell adhesive ability (Yuan et al., 2007). As a member of Ras homolog gene family, RhoC promotes PCa cell invasion and metastasis via sequentially phosphorylating Pyk2, FAK, MAPK and AKT (Iiizumi et al., 2008). Under the effects of Heregulin/HER3‐stimulated signaling pathway, phosphorylated Pyk2 activates the MAPK pathway and facilitates glioma cell invasion (van der Horst et al., 2005). In urothelial carcinoma, FAK depletion doesn't affect (insulin‐like growth factor I [IGF‐I])‐mediated cell invasion while Pyk2 is strongly activated by IGF‐I and promotes IGF‐IR‐dependent motility and invasion. Knockdown of Pyk2 is found to inhibit downstream IGF‐I‐dependent activation of Akt, ERK1/2, p90RSK, as well as ribosomal protein S6K (Genua et al., 2012). Depletion of Pyk2 inhibits tumor necrosis factor receptor superfamily member 19 (TNFRSF19/TROY)‐mediated glioma cell migration by suppressing TROY‐induced Rac1 activity. Pyk2 lies downstream of TROY and plays an important role in TROY‐induced glioma cell migration (Paulino et al., 2010).

### Pyk2 promotes tumorigenesis and tumor angiogenesis by regulating downstream signaling pathways

Pyk2 and FAK are overexpressed in intestinal cancer. Elevated Pyk2/FAK is found to function redundantly in the activation of Wnt/β‐catenin pathway by phosphorylating GSK3β^Y216^ and enhances intestinal tumorigenesis (Gao et al., 2015). In HCC, Pyk2 activates PI3K/AKT pathway to increase vascular endothelial growth factor (VEGF) expression, which is associated with tumor angiogenesis (Cao et al., 2013).

## Concluding remarks

Pyk2 represents a potential high‐value target for therapeutic discovery efforts due to its critical position within signaling pathways, which regulate cancer progression and invasion. Systematical understanding of downstream targets of Pyk2 is necessary to find more effective ways to control human cancer progression. Pyk2 promotes distinct cancers progression by regulating different downstream signaling pathways, and EGFR signaling pathway is found to be involved in Pyk2‐regulated downstream signaling pathways in liver cancer, breast cancer, lung cancer, MM, prostate cancer, bladder cancer, SCCHN, and glioma. Pyk2 could regulate AKT, STAT3, ERK, MEK1/2, Src, HER3, MAPK, EGFR, and STAT5b in EGFR signaling in different cancer types. Moreover, EGFR signaling is reported to be responsible for the single‐agent limitation of FAK inhibitors. As a member of FAK family, the relationship between Pyk2 and EGFR signaling pathway requires more attention in cancer development.

Nowadays, targeting a single receptor using monotherapy often relapses due to the utilization of autonomous parallel‐redundant signaling (Fan and Guan, 2011). There are usually downstream molecules, which enable EGFR‐independent activation to compensate the inhibition of intracellular signaling cascades (Normanno et al., 2009). Approaches to the therapeutic discovery of small molecules, which prevent protein‐protein interactions between key signaling effectors, represent a promising area of anti‐cancer therapy. Combination therapies using two or more drugs usually lead to better anti‐cancer effects. For example, dual inhibition of FAK and Src enhanced the rate of detachment and apoptosis of colon cancer cells than FAK inhibition alone or Src inhibition alone (Golubovskaya et al., 2003). The combination of FAK/Pyk2 tyrosine kinase inhibitor (PF‐562,271) and sunitinib could inhibit different aspects of angiogenesis and tumor aggressiveness and it might have better anti‐cancer effect than a relevant single agent in HCC (Bagi et al., 2009). Pyk2 acts as the crossroad of multiple carcinogenic signaling pathways and Pyk2 is involved in the modulation of EGFR signaling pathway, which facilitates cancer cell proliferation, survival, migration, invasion, metastasis, and chemo‐resistance. In some cases, Pyk2 could regulate malignant biological behavior of tumor when FAK doesn't work (Roelle et al., 2008; Kamihara et al., 2016). Thus, Pyk2 could be considered as an important target in cancer treatment. Significant progress in the exploration of Pyk2‐regulated mechanisms during cancer formation and progression will provide a robust list of potential targets for therapeutic intervention. The combination treatments of Pyk2 inhibitors with molecules that target carcinogenic EGFR signaling pathway may help acquiring better clinical outcomes of anti‐cancer treatment. Some catalytic inhibitors of the FAK, such as PF‐562,271, can also inhibit Pyk2 activity (Bagi et al., 2008). Dual inhibition of FAK and Pyk2 can be more promising in cancer treatment. However, it may be easier to lead to limitation of therapy due to the possibility of activation of complementary EGFR signaling. As there is no relevant study reporting mechanisms of Pyk2 resistance yet, combination treatments of Pyk2 inhibitors and EGFR signaling inhibitors may be a better choice than the combination of FAK inhibitors and EGFR signaling inhibitors in some cases. Moreover, the combined inhibition of Pyk2 and EGFR signaling may be a rescue therapy when the combination treatments of FAK inhibitors and EGFR signaling inhibitors fail to get the ideal effects in cancer treatment.

## Conflicts of Interest

The authors declare that they have no conflict of interest.

## Compliance with Ethical Standards

This article does not contain any studies with human participants or animals performed by any of the authors.
